# Facilitators and barriers in the formulation and implementation of tobacco control policies in Kenya: a qualitative study

**DOI:** 10.1186/s12889-018-5830-x

**Published:** 2018-08-15

**Authors:** Shukri F. Mohamed, Pamela Juma, Gershim Asiki, Catherine Kyobutungi

**Affiliations:** African Population and Health Research Center (APHRC), Nairobi, Kenya

**Keywords:** Tobacco, Control, Policies, Barriers, Facilitators, Formulation, Implementation, Kenya

## Abstract

**Background:**

Tobacco use has serious public health implications for both smokers and non-smokers and significant economic implications on health care spending for governments. Tobacco-related deaths are preventable through well-formulated and implemented tobacco control policies. Using tobacco policy as a case study, we aim to describe the tobacco control policy formulation and implementation and the associated facilitators and barriers in Kenya.

**Method:**

We used a case-study methodology to integrate two sources of data: a document review of relevant policy documents, published articles and reports between 2004 and 2015 (*N* = 24 documents) and in-depth interviews (*N* = 39). Participants were from sectors relevant to tobacco control: research and academia, government, private industry, civil society and non-governmental organizations. Thematic analysis was used to analyze all data.

**Results:**

Kenya developed a comprehensive tobacco policy in 2007. The main facilitators to the policy formulation and implementation process were (1) political commitment and strong leadership, (2) the presence of a coordination mechanism, (3) stakeholder passion and commitment, (4) resources and (5) constitutional requirement for inclusion of stakeholders. The main barriers to policy formulation and implementation were (1) industry interference, (2) resources, (3) poor enforcement and (4) lack of clear roles.

**Conclusion:**

Although the process for formulating a tobacco control policy in Kenya was protracted, the current policy aligns well with current global efforts. The implementation is still weak and this can be enhanced by provision of necessary resources and continued engagement of all relevant stakeholders. There is a need for continued engagement with political leadership and continuous international information exchange on how policy-makers can address and counter industry interference in tobacco control efforts.

## Background

Tobacco use is a major public health priority and a preventable cause of disability and premature death [1]. It increases the risk of various cancers [2], lung diseases, cardiovascular diseases [3], low birth weight, and stillbirth and is therefore a major contributor to premature death worldwide. In 2015, more than a billion people smoked tobacco globally. About 6 million preventable deaths occur annually due to tobacco use [4]. In 2004, tobacco use was estimated to be responsible for 5% of non-communicable diseases (NCD) related deaths in Africa [1], while in 2012, 3% of all deaths among adults aged 30 years and above were attributed to tobacco use in the Africa region [5]. The prevalence of current tobacco smoking is an important predictor of the future burden of tobacco-related diseases. If current tobacco use trends continue, deaths are expected to increase to 10 million deaths each year, and the majority of these deaths will be occurring in low- and middle-income countries (LMICs) [6, 7].

While tobacco use has been either declining or stable in the developed world, it has been increasing in LMICs mainly because the tobacco industry has been aggressively marketing its products in these countries. There is already substantial evidence that all forms of tobacco products cause health problems and result in premature death or disability [1], yet many countries in Sub-Saharan Africa (SSA) have not implemented tobacco control policies to halt the increasing prevalence of tobacco use. International tobacco control efforts have led to ongoing strategies to develop and update existing tobacco control policies in Africa [8].

The World Health Organization Framework Convention on Tobacco Control (WHO FCTC) provides a plan for halting the tobacco use epidemic worldwide. This framework was the first global public health treaty that aims “to protect present and future generations from the devastating health, social, environmental and economic consequences of tobacco consumption and exposure to tobacco smoke” [7]. This treaty was adopted by the 56th World Health Assembly in 2003, and currently 180 countries are signatories to the treaty. The global target to reduce the health threat from tobacco use is a 30% relative reduction in the prevalence of current use in those aged 15 years and above between 2005 and 2025. In order to achieve this reduction, countries can prioritize tobacco control by adopting and implementing the recommended provisions of WHO FCTC, including the implementation of the evidence-based, cost-effective interventions—the “best buy” interventions— tax increases; smoke-free public spaces; ban of tobacco advertising, promotion and sponsorship; and health information and warnings.

Global evidence demonstrates that implementation of strong tobacco policies can reduce the prevalence of tobacco use [9, 10] as well as reduce tobacco-related disease. There is also evidence from a few African countries to suggest tobacco control policies are successful in reducing tobacco use prevalence. Increase in the tobacco excise taxation in South Africa has shown health gains such as reduced cigarette consumption, especially among the poor and young [11]. Mauritius, described as a regional leader in tobacco control in Africa and a world leader in certain tobacco control provisions, began implementing tobacco control legislation in 1999 [8]. Since then, results from several NCD surveys conducted every 5 years show an overall decrease in tobacco use prevalence [8, 12, 13]. With this knowledge on the threat of tobacco use situation around the globe, it is important to have strong tobacco control policies in order to reduce the use of tobacco products and ultimately improve health for all. Despite the signing of global commitments and evidence of successful tobacco control interventions in SSA, few countries have been successful in implementing [5] tobacco control policies according to the WHO FCTC implementation report on the Africa region. The aim of this study is to describe the tobacco policy formulation and implementation and identify associated barriers and facilitating factors to inform and guide future NCD policy frameworks in Kenya.

## Methods

### Study design

In this paper we use a case study design, an approach that facilitates an inquiry of a phenomenon over a period of time using a variety of data sources in order to identify qualities that are true [14]. Document reviews provided background and historical context [15] and key informant interviews with stakeholders were conducted to obtain information about events and to understand participants’ perspectives on policy formulation and implementation.

### Document reviews

Databases were searched to find studies on tobacco control policy documents in Kenya. We searched in PubMed and Google using search terms with different combinations of key words [tobacco control policies + Kenya + tobacco use + barriers + facilitators]. We considered all documents dated 2004 to 2017 to give us a more comprehensive search to include documents from the time Kenya signed the WHO FCTC. Online websites of government institutions such as ministries of Health, Agriculture, Transportation and Education were also searched. We also conducted a manual search in government libraries and offices of government officials targeting documents such as acts, laws, strategic plans, guidelines and government directives. A data extraction sheet was used to collect information about the policy, year of publication, policy objectives, lead actors in the policy process, WHO “best buy” interventions addressed and multi-sectoral approach elements such as stakeholder involvement. This data extraction search was conducted independently by two researchers. Results were compared, reconciled in areas of disagreement, and integrated.

### Key informant interviews

The document review guided the selection of the key informants. Key informants were invited to participate if they were listed in the documents as actively participating in the policy-making process or, if based on the document review, they were reasonably expected to have participated in the process.

The initial study participants were purposefully selected to provide variation in organizational representation and role in policy-making. Additional key informants were also identified using a snowball technique [16] during interviews with the initial key informants. In the event that individuals involved in the policy-making/implementation were no longer in their positions, we made every attempt to contact and interview them. In total, 39 key informants from different sectors were interviewed. The information captured in the key informant interview guide included tobacco policy development, context of the policies, content of the policies, actors in the policy development, policy implementation, barriers and facilitators to the policy formulation and implementation and recommendations or suggestions on how to improve the formulation and implementation process.

### Data collection

We conducted face-to-face interviews with key informants from both public sectors and private organizations that work in sectors relevant to NCD prevention or tobacco control in Kenya, particularly those associated with tobacco control policy formulation and implementation. The sectors included were health, agriculture, education, media and communication, trade, law enforcement, private industry and finance.

Data collection took place between May and December 2014. Interviews were conducted using a semi-structured interview guide. The interviews took place in the key informant’s office or a private place of their preference. On average the interviews took 60–90 min to complete. All interviews were audio recorded and transcribed verbatim to Word files.

### Data analysis

Data were analyzed through thematic content analysis [17]. Walt and Gilson’s policy triangle framework, focusing on four fields: content, context, process and actors who have a critical role in forming policies [18]. Interview transcripts were entered into NVivo 10 software which aids in managing data ideas, data queries, data visualization and data reporting [19].Two researchers independently checked coded 10% of transcripts (*N* = 4) and found 83% inter-coder reliability, which was sufficient [20].

### Ethical considerations

This study was approved by the Research Ethics Board of Kenya Medical Research Institute. Participants were informed about the nature of the study, its potential risks and benefits, confidentiality and their right to withdraw at any time without penalty. Participants were provided with written consent to participate in the study prior to interviews.

In the following section we present the study findings. First we describe the data sources. Then we describe the tobacco control policy using the Walt and Gilson framework. Lastly, we describe facilitators and barriers in the formulation and implementation of the tobacco control policies in Kenya. Information from key informants is italicized and identified by its source.

## Results

### Data sources

#### Documents reviewed

We identified five national tobacco policy documents, four published articles, and 15 reports on tobacco control (See Table 1).

**Table 1:** List of documents analyzed

Document type	Document title	Author	Year
Policy Documents	Finance Act 2012	Government of Kenya	2012
	National Tobacco Control Action Plan 2010–2015	Ministry of Public Health and Sanitation	2010
	Tobacco Control Act	Government of Kenya	2008
	Training Manual on Enforcement of Tobacco control Act 2007	Ministry of Health	2007
Journal articles	An overview of tobacco control and prevention policy status in Africa	Muhammad Jami Husain, Lorna McLeod English, and Nivo Ramanandraibe	2017
	Adherence to the Tobacco Control Act, 2007: presence of a workplace policy on tobacco use in bars and restaurants in Nairobi, Kenya.	Karimi KJ, Ayah R and Olewe T1.	2016
	Tobacco control research in Kenya: the existing body of knowledge	Gladwell Koku Gathecha	2014
	BAT and Public Policy in Kenya, a research paper	Patel, Collins & Gilmore	2006
	Socio-demographic factors of pupils who use tobacco in randomly-selected primary schools in Nairobi province, Kenya	Ogwell A, Astrom A, & Ohaugejorden O	2003
Reports	Kenya STEPwise survey for Non-Communicable Diseases Risk Factor 2015 Report	Ministry of Health, Kenya National Bureau of Statistics & World Health Organization	2015
	The WHO Framework Convention on Tobacco Control: 10 Years of Implementation in the African Region	World Health Organization	2015
	Kenya National strategy for the Prevention and Control of Non-communicable Diseases 2015–2020	Ministry of Health	2015
	Kenya Global Tobacco Survey 2014	Ministry of Health; Tobacco Control Unit & Kenya National Bureau of Statistics	2014
	Kenya Demographic Health Survey 2014	Kenya Bureau of Statistics (KNBS)	2014
	Global Youth survey	Ministry of Health	2013
	“Tobacco Industry Interference in Kenya: Exposing the Tactics”	Ministry of Public Health and Sanitation (MOPHS) & Institute of Legislative Affairs (ILA)	2013
	Shadow Report on the Status of Implementation of the Framework on Tobacco Control (FCTC) in Kenya, 2013	Institute of Legislative Affairs	2013
	Tobacco Control in Africa: People, Politics and Policies	Jeffrey Drope	2011
	Economics of Tobacco Taxation in Kenya	Institute of Legislative Affairs	2011
	Situation Analysis of Tobacco Control in Kenya: Report of the Baseline Assessment carried out by Kenya Tobacco Control Situational Analysis Consortium	Kenya Tobacco Control Situational Analysis Consortium	2008
	Kenya Demographic Health Survey 2008–2009	Kenya Bureau of Statistics (KNBS) and ICF Macro	2010
	Global Youth survey	Ministry of Health	2007
	Kenya Demographic Health Survey 2003	Central Bureau of Statistics, Ministry of Health and ORC Macro	2004
	WHO Framework on Tobacco Control	World Health Organization (WHO)	2003

#### Key informant interviews

Interviews were conducted with 39 individuals from the different sectors (See Table 2). The majority of participants were from the health sector, particularly from the Kenya Ministry of Health (MOH) though they were from different units within the Ministry of Health. For instance, there were participants from Ministry of Health who represented the NCD, enforcement and the nutrition units.

**Table 2:** Number of respondents interviewed by sector

	Government including parastatals and academia	CSOs including CBOs, NGOs	Bilateral organizations	Private sector (including industry)	Total
Health	8	3	1	1	13
Agriculture	1	1	2	0	4
Education	1	0	0	0	1
Media and communication	0	0	0	1	1
Trade	2	0	0	0	2
Law enforcement	1	0	0	2	3
Parliament	3	0	0	0	3
Finance	1	0	0	0	1
Other sectors	5	5	0	1	11
Total	22	9	3	5	39

#### Tobacco policy context

Results from the document review and the key informant interviews revealed a combination of strong global initiatives, local epidemiological factors, economic and political factors that contributed to tobacco policy formulation.

##### Global context

From a global context, the development of the current Kenyan tobacco legislation (the Tobacco Control Act) was largely influenced by the FCTC adopted in 2003 at the World Health Assembly [21]. The bilateral official quoted below indicated that Kenya largely adopted the components of the FCTC.

*…when you sign and ratify [the WHO FCTC], you are obligated to do what the framework says. Kenya is one of the countries that had signed and ratified [it] and therefore it was obligated to make sure that it domesticates the treaty. That’s why the [Tobacco Control Act] is actually just the mirror image of the FCTC. –Bilateral Official 3*

##### Local/epidemiological context

Although tobacco policy formulation efforts were largely driven by international efforts, epidemiological factors also played an important role in Kenyan tobacco policy development. According to the key informants, tobacco use was widely considered a public health concern and there was a heightened awareness that tobacco use causes more harm than good to the population and the general environment.

*[At the time of the initiation of the tobacco control legislation,] people were seeing [tobacco] as a public health issue. People were seeing it as an issue that was affecting them, affecting the masses. –Academia Official 1*

Although the focus of the WHO FCTC is health, one Ministry of Health official also identified environmental impacts as a concern.

*In addition to the disease or health effects of tobacco, we also have the environmental effects, especially in tobacco-growing areas where it causes deforestation, the leaching of the soil, contamination of the water, pollution of environment, the soil the water and the air.* –*MOH Official 5.*

Despite participants’ concerns about the public health impact of tobacco from both local and global studies, some felt there was not enough up-to-date epidemiological data to provide a context for policy development. This data gap was mentioned as a barrier to characterizing the tobacco problem.

*The other big gap we have is data. When you are trying to do research on NCDs you realize that the biggest challenge you will have is data. How many people really drink alcohol? How many people are smokers? etc*. *–MOH Official 3*

##### Economic context

Document review of economic analysis showed that the cost of treating tobacco-related illnesses was higher than the monetary benefits the government was receiving from tobacco sales taxes [22]. Similarly, many of the key informants consistently reported more funds are spent on treatment compared to the revenue that the tobacco industry generated for the country despite claims to the contrary by the tobacco industry. Despite the money it generated, tobacco use in Kenya exerted a considerable health burden on the economy. This Ministry of Health official identified the cost-benefit ratio for Kenya as highly in favor of tobacco control.

*We get about 6 billion [Kenyan Shillings] or so in terms of earnings, or let’s say, simply put, for every dollar we get from the tobacco taxation, we spend about three dollars trying to sweep whatever mess it has caused in terms of cancers, in terms of environmental degradation, in terms of destroying our water catchment areas. Tobacco causes a lot of damage, so now we understand that there is less to gain from the industry. In terms of money, coin for coin, we are losing. –MOH Official 3*

Respondents said that the tobacco industry exaggerated the extent to which they pay taxes and provided false information about employment they create to deliberately sow confusion in the public.

##### Political context

Lastly, respondents described strong political influence from the highest level as providing the impetus in 2004 for signing and ratification of WHO FCTC bill. The final push came in 2004 when the First Lady toured a hospital’s pediatric cancer ward and was touched by the children’s cancer cases. Immediately following her tour, she arranged for the placement of the WHO FCTC as an agenda in a cabinet meeting for approval. Surprisingly—and a first for Kenya in terms of ratifying an international convention—the cabinet approved the WHO FCTC, and it was signed and ratified the same day. The Tobacco Control Act was signed into law in 2007. This was unexpected and was likely associated with it being an election year. Thus the WHO FCTC’s strong and binding properties played a major role in catalyzing the process of developing the tobacco control act in Kenya.

### Tobacco policy content

Currently, the Tobacco Control Act 2007 [23] is the principal law governing tobacco control in Kenya. This comprehensive law covers all topics recommended in the WHO FCTC, including directly addressing the “best buys” interventions of tax increases; smoke-free public spaces; ban on tobacco advertising, promotion and sponsorship; and health information and warnings. Key elements in the tobacco control policies in Kenya are summarized in Table 3.

**Table 3:** Elements of tobacco control policies in Kenya

Policy /Year	Elements in the policies
Tobacco Control Act 2007 (came into effect 2008)	Restricts smoking in public places Prohibits tobacco advertisement, promotion and sponsorship Requires health warnings and messages on tobacco products Recommends raising tobacco excise taxes to 70% of retail prices Enforcement
The National Tobacco Control Action Plan 2010–2015	Outlines public health policy on tobacco control for Kenya and Facilitates implementation of key recommendations of the TCA
Finance Act 2012	Raises excise duty rates on tobacco products
Tobacco Control Regulations (tabled 2014)	Requires cigarette packaging to have no brand names or trademark Requires cigarette packaging to have health warnings and visual pictograms in color

After the Tobacco Control Act was passed, a training manual on its enforcement [24] was developed in the same year. To facilitate the implementation of the Act’s key recommendations, the National Tobacco Control Action Plan 2010 [25] was developed. This Plan focuses on a number of evidence-based interventions drawn from the WHO FCTC and the Tobacco Control Act 2007.

In early 2012, the Finance Act 2012 was published in an official gazette. It sought to raise excise duty rates on tobacco products. In December 2014, the Tobacco Control Regulations 2014 were published. These regulations were to become enforceable 6 months after the gazette notice, intended to strengthen the WHO FCTC and the Tobacco Control Act 2007 implementation. However, in 2015, the high court in Kenya ordered the regulations to be temporarily suspended until a British American Tobacco lawsuit alleging the constitutional requirements were not followed in the regulation-making process was resolved. After more than a year, a ruling was made in favor of the Tobacco Control Regulations 2014 and the regulations went into effect in September 2016. Despite British American Tobacco’s filing an appeal against the High Court judgment, the court ruled that the tobacco company’s appeal had no merit and affirmed the decision of the lower court.

Key informants indicated that they were satisfied with the rigorous approach of the Tobacco Control Act and subsequent Acts. They commented that Kenya’s tobacco policies comprehensively addressed components of the WHO FCTC such as demand reduction, supply reduction, awareness creation, prohibition of illicit trade, taxation of tobacco products, creation of smoke free environments and a total ban on smoking in public areas.

### Policy process

#### Agenda setting and policy formulation

According to document reviews, discussions about Kenya’s tobacco policy agenda began in 1992 when it participated in World Tobacco Day campaigns. Some of the respondents mentioned that the first tobacco control bill was drafted in 1998 before the WHO FCTC ratification occurred. The 1998 Tobacco Control Bill was described as very weak in terms of content because it was drafted before the WHO FCTC. After the legislation was drafted, the Ministry of Health established a National Tobacco Free Initiative Committee in 2001. In 2003–2004, the focus shifted to ratification of the WHO FCTC. Once it was signed and ratified in 2004, the domestication of the FCTC began in Kenya. Bills were then prepared and presented in parliament every year, but adopted only in 2007.

Other parallel policies affecting tobacco control such as the tobacco taxation policies were in effect at different times, even as a comprehensive tobacco control policy was in development. The policy process was rather ad hoc and iterative, with parallel policies in development and implementation at different times. For instance, tobacco taxes were levied before the Tobacco Control Act was formulated. Reforms in tax policy also took place separately from the process culminating in the Tobacco Control Act. In addition, alternative cropping policies in the agriculture sector encouraged farmers to reduce tobacco growing. The push for a comprehensive policy was partly a result of obstacles encountered while enforcing aspects of the older law, Public Health Act (CAPS 242, 1982 & 1990). A comprehensive policy was needed to support these measures and to expand enforcement efforts beyond the large municipalities. The Tobacco Control Act’s formulation and subsequent amendments were an opportunity to harmonize these policy initiatives.

The tobacco policy process—that is, the policy to develop a comprehensive Tobacco Control Act as well as a technical policy—gained momentum after Kenya signed and ratified WHO FCTC in 2004. In addition, the process of getting the technical policy led by the responsible line ministry worked in reverse: the legal instrument needed to support enforcement of the technical policy came before the Act was enacted and before a comprehensive tobacco control policy or action plan were in place anywhere in government.

Participants said the tobacco policy process was challenging and took a long time before it was enacted into law. However, poor documentation of the process meant that most respondents could not recall the steps they went through during the Tobacco Control Act’s formulation. The resistance from the tobacco industry was strong and their interference could partly explain the delay in formulating the Act and actual development of a comprehensive tobacco control policy. Lack of funding was also cited as a reason for the slow pace at which the policies were formulated.

### Policy implementation status

Participants’ views and document reviews indicate that some of the provisions of the Tobacco Control Act 2007 have been implemented, and most of the WHO “best buy” interventions for tobacco control have been implemented to some extent.

#### Tax increases

There have been tax regimen changes since the Tobacco Control Act 2007 came into effect. Recently, the Finance Act raised the excise duty on tobacco products at a rate of KSH 1200 per 1000 or 35% of the retail selling price, although it is still lower than the WHO tax recommendation of 70%.

#### Smoke-free public spaces

Tobacco use in all public places is prohibited and enforced in most areas [23, 26].

#### Ban of tobacco advertising, promotion and sponsorship

Tobacco advertisement, promotion and sponsorship of all forms is also prohibited by the Tobacco Control Act. However, outdoor advertisements on billboards and buildings were still seen in several parts of the country [27].

#### Health information and warnings

Health information and warnings have been implemented for tobacco packaging. These texts now cover 30–50% of the front and back display of tobacco product packages.

#### Training

The government has also undertaken training of enforcement officers, media and civil society organizations to support the implementation efforts. Increasing awareness due to the trainings has led to removal of billboards with tobacco brand advertisements in major cities.

#### Monitoring and evaluation

Implementation is now focusing at sensitizing the decentralized government structures (county level) on the Tobacco Control Act requirements within the law. Training has also been conducted to guide enforcement agents. The country is also making a strong effort to collect better epidemiological data to better ascertain the impact of the policy changes; questions on tobacco use have been added to several surveys including the Kenya Stepwise survey 2015, Kenya Demographic and Health Survey (2003, 2008–09 and 2014), Global Tobacco Youth Surveys (2007 and 2013) and Global Adult Tobacco Survey (2014). The 2008 and 2014 Kenya demographic health surveys have indicated no improvement in the prevalence of tobacco smoking among adults aged 15–49 (19% vs. 19%) but there was an improvement when compared to the 2003 Kenya demographic survey (23% vs. 19%). The youth survey has revealed an increase in overall tobacco use among children aged 12–15 years within a period of 6 years (13% in 2007 vs. 18.6% in 2013).

### Actors

The policy process was consultative, involving multiple government sectors and other stakeholders. The Act established the Tobacco Control Board consisting of various sectors relevant to tobacco control including a Ministry of Health member. The Ministry of Health and the Tobacco Control Board led the policy process, with civil society organizations strongly engaged in the policy-drafting stage.

Informant interviews revealed participation of actors from different health and government sectors including non-governmental organizations, community based organizations, civil society organizations and the private sectors. Table 4 summarizes the roles played by the different institutions, sectors and stakeholders mentioned in the study in addition to reviewing the policy drafts.

**Table 4:** Roles of actors in the tobacco policy development process

Institutions/Sectors/Stakeholders	Roles in policy formulation and implementation
Ministry of Health	Led formulation and implementation processes, spearheaded policy process, drafted policy documents, trained enforcement officers and health workers to implement TCA, facilitated government departments to implement their roles, provided technical expertise to stakeholders involved in policy formulation, fundraising
Ministry of Finance	Provided information on revenue amounts generate from industry; engaged in taxation discussions
Ministry of Agriculture	Provided suggestions to tobacco growers on alternative cropping, results from soil economic analysis studies on which crops would give higher yields
Ministry of Education	Contributed to policy process; expected to protect and educate children and include this in their school education/health policy
African Medical and Research Foundation (AMREF)	Provided research findings on tobacco studies; contributed to the process
Ministry of Trade	Increased taxes on tobacco; reduced illicit cigarettes
East African Community (EAC) ministry	Ensured harmonization of trade and tax regimens across East Africa to avoid illicit trade of tobacco products
Tobacco industry	Provided information on product contents
Kenya Revenue Authority	Involved in process to track tobacco products for tax purposes
Pubs, Entertainment and Restaurant Association Kenya (PERAK)	Participated in process to provide industry with guidance on how law/policy compliance
Police	Implemented law, followed up on contraband tobacco products
Children’s department	Provided information on early initiation of drugs in schools
Ministry of Environment	Provided environmental laws on environmental pollution and environmental degradation due to tobacco curing
Kenya Medical Research Institute	Provided research, participated in the meetings, contributed to bill content and led baseline situational analysis
Attorney General’s office	Advised on regulations with reference to legal requirements of a policy document; legal advisors to the government; assisted drafting and implementation of legislation; advised on bill structuring and statues before law could be drafted
Law Society of Kenya	Legal entity representing the general public, also acts as government watchdog. Supplemented attorney general perspective and advice and verified AG prescriptions in line with practice norms
Business community (Kenya Association of Manufacturers)	Employs a large number of population
Consumer information network	Organization that empowers consumers through education and advocacy, research on consumer concerns and to effectively serve as a center of integrity on consumerism
NGOs – Kenya Tobacco Control Alliance	Drafted TCA; assisted in outreach such as public education, rallies and processions; raised awareness via social media; conducted research and disseminated findings; monitored industry violation and interference; monitored Ministry of Health and enforcement officers to ensure duties were carried out; monitor CSO implementation; nationwide training of enforcement officers and local leaders; participated in county-level legislation process; assisted drafting county-level regulations; asked Kenya to take part in international anti-tobacco process
Local government (provincial administration)	Implementation work, for example: designation of smoking zones, destruction of the tobacco advertising billboards
CSOs – civil societies – Institute of Legislative Affairs	Advocated for tobacco control; lobbied; funded meetings; monitored compliance (enforcement); mobilized people and supporters; raised awareness
Bilateral organization (WHO)	Provided technical support; advised on policy; suggested what to emphasize; ensured proposals were line with FCTC guidance; provided funding
Academia	Provided research findings; justified policy’s importance; compared international tobacco policies
Other international organizations (US, Denmark, etc.)	Provided information on how anti-tobacco law implementation is successful in their countries
Members of Parliament	Supported the bill; assisted pushing bill through parliament
Media	Messaging

### Facilitators to tobacco policy formulation and implementation

Several key facilitating factors have been described in the tobacco policy formulation and implementation:

1. *Political commitment and strong leadership*. The Ministry of Health played a big role in taking the lead, but a strong civil society was mentioned as critical to initiating and sustaining the process. The Ministry of Health leadership engaged many stakeholders who assisted in drafting the policies, providing input at meetings and guiding the process to meet WHO FCTC guidelines. Study participants felt stakeholders needed to be engaged early in the process otherwise it would be difficult to get their buy-in or it would take a long time to bring them up to speed with all the earlier discussions and decisions.
  *I think there was a common understanding among people in the movement against tobacco. We did not have a challenge of their coordination because the Ministry of Health took the stewardship of guiding them. It was government leading the way and they were coming in to contribute.* —*Ministry of Health Official 4*
  *My experience has been that it requires a lot of political good will from the government … political commitment to the issue, that’s number one. Number two is a strong coordination mechanism that ensures at the end of the day that everybody participates, everybody owns the process, and everybody contributes to the process, so that you don’t have stakeholders who feel that they are passengers on this process.* –*CSO/NGO/CBO Official 3*

The participants noted that with the decentralized government system in Kenya, county-level goodwill and leadership is key to implementing the tobacco policy. They also noted that most counties had started developing county-specific tobacco policies, and some trainings were also occurring now at the county level.

(2) *Presence of a coordination mechanism*. Having many stakeholders on board meant that a stakeholder coordination mechanism was necessary to guide the policy process. Thus a coordination mechanism was described to be a facilitating factor during the formulation process. Respondents mentioned that a National Tobacco Free Initiative Committee (NTFIC) was developed about the same time as the Tobacco Control Act, and it was convened by the NTFIC chairman who heads the Ministry of Health Division of Non-Communicable Diseases. The Director of Medical Services also chaired the committee from time to time. The mechanism served three purposes: information-sharing, discussion of strategy and coordination of activities. The committee was also described as having had a wide representation from the relevant tobacco control sectors, including government, civil society, religious bodies and the private sector. Also, having an accountability mechanism was seen as key as it brought a measure of responsibility.
*I think if we have a coordination mechanism that all players meet, that all players plan together and allocate resources and allocate responsibilities, then we can actually go far in joint planning, implementation and also resource mobilization. –Ministry of Health Official 5*
*The coordination committee (National Tobacco Free Initiative Committee) would meet once a month to discuss and agree on strategy, coordination of the various activities by civil society and government departments (in lobbying, media advocacy, drafting, education and awareness, how to engage with industry, etc.), and allow sharing of information. The team met once a month and if there was something special to discuss, a special meeting would be called. There was no funding to support this at the time and the Ministry of Health availed its board room. –CSO/NGO/CBO Official 7*
(3) *Stakeholder passion and commitment*. Passion and commitment were reported as important to policy development and implementation. These traits were mainly seen in champions or advocates, and they kept the tobacco policy environment vibrant. Respondents also echoed that common goals and understanding, interests and vision were very important factors, and once stakeholders knew what their roles were, it was easier for them to work towards that common goal.
  *It requires a lot of commitment and passion by the tobacco control advocates.* –*Ministry of Health Official 5*
  *Then we have the Tobacco Control Board (TCB), which has individuals and characters who are very passionate … about tobacco control. So the environment has been kept vibrant because of the passion of the players. Also the TCB, which the act gives an advisory role, has really done their part.* –*Ministry of Health Official 3*
  *It is the general consensus that tobacco is harmful to human health, and that we need to control tobacco consumption. That was a basic understanding, and therefore, the act was hatched, and now it is a law*. –*Other Ministries Official 3*
  *A lot of people agree tobacco is harmful. I mean, it is not like alcohol, where you have to convince the policymakers that we should regulate this. We have reached a point where we have seen now people coalescing around tobacco control.* –*Ministry of Health Official 3*
(4) *Resources*. Resources to support the policy formulation and implementation process were described to be a key facilitator. These resources include funding and expertise from both the government and the international community. A clear budgetary allocation to support activities was also noted to be a facilitator. Funding was received for different policy formulation activities from other stakeholders, not the government. For instance, study participants reported that WHO, Civil Society Organizations (CSOs) and NGOs supported the tobacco policy process by providing funding for meetings, travel and other activities.
*The Ministry of Health … has been very supportive both in terms of resources and expertise, so we have worked together so well. The other one is a lot of support from the international community, because most of the activities we have undertaken have been funded by partners from outside. –Civil Society Organization/Non-Governmental Organization/Community Based Organization Official 4*
(5) *Constitutional requirement for inclusion of stakeholders*. Another facilitator was Kenya’s constitutional mandate requiring consultation of all stakeholders. As the Tobacco Control Act subsidiary policies were developed after the new constitution was in place, participants had to bring all stakeholders together, including the tobacco industry, to fulfil the constitutional requirement.

### Barriers to tobacco policy formulation and implementation

Many challenges were described by the study participants during the formulation and implementation of the tobacco control policy. Tobacco industry interference was a key barrier to the tobacco policy process.

1. *Tobacco industry interference*. Unsurprisingly, the tobacco industry was responsible in the poor implementation of Kenya’s tobacco control policies. Their interference included bribery, violations of existing policies and challenges to the roll-out of new policies and amendments. Participants cited several examples of the industry violating advertising provisions and peddling influence. Tobacco manufacturers continue to advertise their products in local newspapers despite banning of this practice. Participants noted that tobacco policy development and implementation took so long because the industry used delaying tactics to slow the process down, such as instituting a legal suit to delay the printing of the health warnings on tobacco products or compromising officials with bribes like luxurious holidays and retreats for the ministry employees. This lengthy process was noted to have deterred some actors from engaging with the tobacco control team.
  *Industry interference makes it very hard … Like now, the industry has been violating the law by advertising in the papers, they have recently had—I can’t recall the date well, but about one month ago—British American Tobacco placing Embassy [tobacco product] in* The Nation *of a Friday and then* The Standard of Saturday *[largest local daily newspapers] showing the changes that they have made on their product*. –*CSO/NGO/CBO Official 4*
*First of all, tobacco industry has always been against tobacco control not directly because they always claim that they are not but they, they watered down the Tobacco Control Act that we had. The … Act that we have today is not what we had proposed in the first place because they just, they influenced the content that went into the Act. –Ministry of Health Official 4*
*We agree on this as we go away, they [tobacco industry] send a booklet with information contradicting what we have agreed on, and then we start from square (one) again. It went on and on: I am sure you know of how the industry was supporting the politicians because we were developing a bill which should go to the parliament to be debated before it is passed into an act. The industry also went to the politicians. –Bilateral Organization Official 3*

Participants said the tobacco industry was powerful mainly because it has money, which the ministries working on the tobacco polices didn’t have. The industry disguises itself or hides behind pro-tobacco policy NGOs to distort the policy process. Participants also accused the industry of deliberate misinformation to slow down the process. Some felt that NGOs receive funds from the industry and as a result advance the industry’s cause by bringing up arguments in meetings.

Another tactic of the tobacco industry was taking advantage of changes in government by pushing its agenda to the new government even if a decision had already been settled. Another strategy the industry was manipulation of public opinion. This was in the form of a campaign highlighting government unfairness in legislating tobacco control despite the industry being a key employer in an environment of high unemployment.

(2) *Resources*. One of the barriers to tobacco policy development and implementation was lack of both financial and human resources. Lack of funds was cited as the reason behind the long process of getting the policy in place and also the slow progress in implementation. According to a shadow WHO FCTC report by the Institute of Legislative Affairs, the Tobacco Control Fund set up under Tobacco Control Act did not initially receive budgetary allocations from the Ministry of Finance [26] to support implementation of the Act’s provisions. The fact that it was not done in the early phase is a manifestation of the policy’s partial implementation. Respondents also concurred that there was close to nothing set aside for implementing the Tobacco Control Act provisions.

Lack of human resources was also cited as a hindering factor in implementation. The Ministry of Health was reported to have few staff in the tobacco control unit; staff turnover or reassignment also affected the policy implementation. It was unclear whether people are assigned to tobacco control roles in other ministries outside the health sector, which is a reflection of the limited funding allocated to tobacco control generally.

*Another thing is turnover. We have an officer here [who] has a wealth of experience in tobacco control matters—if he leaves, what happens? There is no succession. We need to spread that out so that we have more training, more people—we are very understaffed.* –*Ministry of Health Official 3*

(3) *Poor enforcement*. Another challenge was lack of enforcement by those charged with its responsibility. This lack might be partly attributed to the lack of resources experienced by other players in tobacco control, including the Tobacco Control Board, which was reported not to have received funding since 2009. It might also be a result of lack of awareness by law enforcement agents of their role, given their limited participation in policy formulation.
  *It is the law that has to be enforced and the police are not good in this country in enforcement … now the law is very clear about what needs to be done … but you find people breaking that law and police are just watching. You find guys hawking one cigarette by one, even in the traffic jam, [though] it is illegal to sell cigarettes in sticks. The law says you can only sell in a packet, but now you find people hawking in sticks. You are supposed to put health warnings [on packages] but if you sell in sticks, it means that at the point of sale, you don’t have health warnings. The mechanism for enforcing that is not there ... enforcement is the challenge*. –*Ministry of Health Official 4*
  *Limited resources also interfere [with implementation]. There are times the enforcement officers are unable to move, maybe because they don’t have a vehicle, or they find that these distances are long, it becomes another issue*. *–CSO/NGO/CBO Official 4*
(4) *Lack of clear roles*. A lack of clarity on roles of Tobacco Control Unit within the Ministry of Health and the Tobacco Control Board undermined utilization of the little funding that tobacco control received and led to delayed funding disbursements from the government.
*It is also because of … disorganization in the ministry, there [have] been a lot of problem[s] in the ministry where it is not clear what the board should do, what should the tobacco boss of that unit do … and then there is the non-communicable disease division. What should it do, and yet the boss of the non-communicable diseases section is the secretary to the board? The board looks like it works separately from the division, so that kind of who-should-do-what and who-belongs-where has been a problem and that has made it hard to allocate funds, because it is like, who will control the funds? –CSO/NGO/CBO Official 4*
  *Then they receive some money from donors—the board received some money from donors—but then that money cannot be enough for the activities that are required.* –*CSO/NGO/CBO Official 3*

## Discussion

This study provides insights about the key facilitators and barriers encountered in formulating and implementing the tobacco control policy in Kenya. Kenya has a comprehensive tobacco policy in place, which was developed in 2007 [23]. This policy was enacted 3 years after Kenya ratified and signed the WHO FCTC [28]. Facilitators in policy formulation and implementation were (1) political commitment and strong leadership, (2) presence of a coordinating mechanism, (3) stakeholder passion and commitment, (4) resources and (5) constitution requirement for inclusion of all stakeholders. The major barriers that contributed to a delay in the policy formulation and implementation include (1) industry interference, (2) lack of resources and (3) poor enforcement. These findings are consistent with the global literature on tobacco control policy formulation and implementation.

### Political commitment

The WHO FCTC has declared that strong political commitment is necessary at all levels of tobacco control in order to have a successful tobacco policy process [21]. Experiences from HIV/AIDS programming has shown that high political leadership is critical for action in a multi-sectoral coordination mechanism. This was also evident in Turkey’s successful tobacco control journey where there was strong support from the minister of Health and the prime minister [29]. For Kenya, the two major milestones in the policy development process were achieved through the good will of politicians. First, the Ministry of Health and civil society organizations engaged a political actor just after the ratification of the FCTC through a tour of a cancer ward that enabled the bill to be tabled immediately in parliament. Another politically expedient opportunity came in 2007 (during presidential elections) where the presidency was engaged and the bill was enacted.

### Coordination mechanism

To ensure smooth coordination of tobacco control activities, there is need to establish a mechanism clearly defining roles and duties [28]. A successful coordination mechanism acknowledges the need to assign responsibility and require accountability from the participating sectors and stakeholders in a consultative manner [30]. This will accelerate the policy process and reduce unnecessary delays which are often very demotivating to actors who are keen to see a policy in place. The Ministry of Health in Kenya took a lead role in coordinating the meetings and providing technical guidance to the policy discussions which contributed significantly to policy development.

### Stakeholder passion and commitment

Efforts in promoting policies to curb tobacco use often involve committed individuals who are passionate about tobacco control even when facing strong opponents, such as the tobacco industry, that interfere with the policy-making process. At the celebrations marking 10 years since WHO FCTC went into force, a Jamaican minister of health remarked that “moving the agenda of the FCTC implementation forward also requires passion and determination to be resolute in the face of challenges” [31]. Since stakeholder engagement in policy-making is enshrined in the Kenyan constitution, the Ministry of Health involved several actors and this promoted in-depth policy discussions and stakeholder ownership.

### Resources

Both financial and human resources have been identified to be both facilitators and barriers to tobacco control efforts. Lack of funding hampers progress in implementation in other countries [5, 32]. WHO recommends that tobacco control efforts should be adequately funded at all stages of the process [33]. Additionally, building capacity for tobacco control has been termed to be an urgent priority for successful tobacco control initiatives [33]. Although funding was available from donors for the policy development process in Kenya, policy implementation faced funding constraints, leading to a weak implementation.

### Constitution requirement for inclusion of all stakeholders

The Kenyan constitution provides an opportunity for an inclusive policy process that promotes multi-stakeholder engagement. This was similar to the recommendation made in the WHO Action Plan which recognized the importance of multi-sectoral engagements at all levels for preventing and controlling NCDs [34].

### Tobacco industry interference

Tobacco industry interference tactics are known to disrupt tobacco policy formulation and implementation in many countries and this is well documented [35–38]. WHO has cautioned that tobacco industry interference can take many shapes or forms and may not be obvious, but it is important that all countries are aware of their tactics and take action against them [39]. In Kenya the protracted period from FCTC ratification to implementation of a Tobacco Control Act, resulted from the tobacco industry’s undue interference in form of bribes, violation of existing policies and a legal suit challenging some of the aspects of the policy.

### Poor enforcement

Enforcement is critical to policy implementation yet it often lags behind [33]. Even in countries where policies are in place, poor enforcement has hampered tobacco control efforts. This is consistent with experience in other countries [40]. The law enforcement officers (police) were not adequately sensitized thus unable to fully enforce the tobacco control regulations. Lack of funds needed for capacity building of enforcement officers was also cited as a major setback in implementing the Tobacco Control Act in Kenya.

## Conclusion

Despite these challenges in formulating tobacco control policies, Kenya moved relatively faster than other countries in the region from FCTC ratification to a Tobacco Control Act because of a high-level political support. The Ministry of Health and civil society used two political events to draw the attention of key political figures: first in the signing and ratification of the global treaty and, second, through the first lady’s visit to a pediatric cancer ward, where the technocrats were able to quickly link the burden of cancers to tobacco, thus steering the Tobacco bill on to the cabinet and parliament. Similarly, the election year 2007 was another opportunity to present the Tobacco Control bill for the president to sign. When high-level political support is achieved, there appears to be more momentum in the policy formulation process. It is therefore crucial that those engaged in policy formulation consider engaging political support in the early phases of policy development.

Evidence of good political will is already emerging from Kenya’s decentralized government system. It has been reported that most counties have started developing county-specific tobacco policies and trainings for implementation. This will provide the right impetus to the implementation process.

Kenya’s experience can serve as an example to other countries of what can happen when political will/stakeholder commitment is solid. There is also a need for continuous epidemiological data to initially show tobacco use as a problem and to then monitor progress of tobacco control policies. Many factors highlighted in this study are applicable to low-middle-income-countries that are in the early to middle stages of the tobacco control process. These countries need to be particularly aware of the tobacco industry tactics in attempting to prevent policy development and implementation, as the industry aggressively expands their markets in these countries. Lastly, there is value in documenting the policy processes in real time including sharing lessons from various settings.

## Abbreviations

ANPPA: Analysis of Non-Communicable Disease Prevention Policies in Africa
MOH: Ministry of Health
MSA: Multi-sectoral approach
NCD: Non-Communicable diseases
SSA: Sub-Saharan Africa
TCA: Tobacco Control Act
WHO: World Health Organization
